# Can We Trust Score Plots?

**DOI:** 10.3390/metabo10070278

**Published:** 2020-07-08

**Authors:** Marta Bevilacqua, Rasmus Bro

**Affiliations:** Department of Food Science, University of Copenhagen, Rolighedsvej 26, 1958 Frederiksberg, Denmark; marta@food.ku.dk

**Keywords:** overfitting, score plots, validation

## Abstract

In this paper, we discuss the validity of using score plots of component models such as partial least squares regression, especially when these models are used for building classification models, and models derived from partial least squares regression for discriminant analysis (PLS-DA). Using examples and simulations, it is shown that the currently accepted practice of showing score plots from calibration models may give misleading interpretations. It is suggested and shown that the problem can be solved by replacing the currently used calibrated score plots with cross-validated score plots.

## 1. Introduction

In areas such as metabolomics and proteomics, the PLS (partial least squares) regression model is often used for classification purposes, which is then referred to as PLS-DA. In the following, we will use both names, but the data analytic examples all pertain to classification in this paper. Sometimes PLS-DA is used for creating predictive models, or for visualizing and perhaps identifying biomarkers. In any case, the utility of PLS-DA is apparent. It handles missing data points, correlated variables and datasets with more variables than samples. In addition, it provides excellent tools for visualizing the relation, for example, between observations using score plots. There are also alternative versions of PLS-DA such as OPLS-DA (Orthogonal PLS-DA) which we will all group together in this paper as they share features in terms of when the visualization is of doubtful quality [1].

The quality of the prediction model can be measured by *R^2^*, which is the fraction of variance of the response that is explained for the calibration model samples. When measured using a test set or cross-validation, the number is called *Q^2^*. Sometimes, the quality of a given PLS-DA model is moderate or maybe even weak. That is, the model has little predictive power. This can be reflected in the difference between *R^2^*—how well the model predicts the calibration data, and *Q^2^*—how well the model predicts new data. It is our claim here that when the difference between *R^2^* and *Q^2^* is too large, the score plots from calibration models no longer provide a meaningful visualization of the pertinent information in the model. We will exemplify this and also provide an alternative solution which will obliterate the problem.

## 2. Theory

For descriptions of PLS and OPLS, we will refer to the literature [2,3]. For a description of the use of PLS for discriminant analysis, see [4]. In essence, (O)PLS-DA is the same as a standard (O)PLS model using a specific response, which is a dummy variable indicating belongingness for each class. Both PLS and OPLS provide a model of the data, **X** (*I×J*), used to predict the response **y** (*I×*1). The model of **X** can be written as:

**X** = **TP**^T^ + **E**, (1)

where **T** is a (*I×F*) score matrix and **P** a (*J×F*) loading matrix of an *F*-component model. The model of **y** is given:

**y** = **Tb** + **e**, (2)

where **b** is the so-called inner relation regression vector of size *F×*1. In the equations, we disregard the preprocessing of the data for simplicity and also assume only one dependent variable in **y**. It is the score values in **T** that are often used for score plots. Often component one and two are plotted against each other. This makes particularly sense for the OPLS, where it is known that the first component will hold the predictive information.

Sometimes models are overfitted; for example, if the **y**-relevant signal in **X** is very weak or maybe even absent. Then, it is common to observe that the predictions of the samples that are part of the calibration set that are much better described than new samples e.g., those predicted during cross-validation. Hence, in these cases, *R^2^* will be much bigger than *Q^2^* (*R^2^* >> *Q^2^*). There are ample descriptions of this in the literature [5,6,7].

When *R^2^* >> *Q^2^*, the parameters of the model can be severely influenced by noise or other irrelevant information. Hence, for example, the calibrated scores will tend to reflect the variation of **y** much more than the model predictively will—they will be optimistic. This holds for PLS as for OPLS. It is expected that for OPLS this effect can be larger especially for models that have many components. For one- or two-component models, it is expected that score plots from PLS and OPLS will overfit to the same degree, essentially.

Instead of using scores from a calibration model known to be overfitted, it is possible to use validated scores, obtained for example from cross-validation. These cross-validated scores will not suffer from overfitting as is the case with the scores from the calibration model. However, due to the rotational freedom of bilinear models, it is necessary to correct the scores for sign and rotational ambiguity to avoid overly pessimistic scores. There are several possible approaches to that as outlined e.g., in [8].

Here, we adopt the following approach. An overall model using all calibration data is obtained as given in Equation (1). The loading matrix of this model defines the target configuration. In every cross-validation segment, the loading matrix of the PLS model obtained from the smaller dataset is rotated towards the loadings of the overall model. This can be done either orthogonally or obliquely and as mentioned also in other places, the choice of which to use is debatable [8]. In initial tests, there was little difference between the two approaches and both clearly removed the obvious meaningless differences related, for example, to sign switches. Hence, the effect of which rotation to use was negligible compared to the effect of doing rotation. Oblique rotations are used in the following. The rotation matrix obtained from the rotation can be used to counter-rotate the scores of the cross-validation models. In addition, this counter-rotation can also be applied to the scores of the left-out sample(s). This way, we obtain rotated scores for each left-out sample and, after a complete cross-validation, we have a validated score matrix that can be compared with the calibrated scores matrix.

## 3. Data

All the data and data analysis were performed in MATLAB using the PLS_Toolbox version 8.8.1 [9]. See the Supplementary Materials for the scripts used to produce plots in the article. To build the classification models, PLS-DA and OPLS-DA was used.

### 3.1. Simulated Data

**Figure 1:**
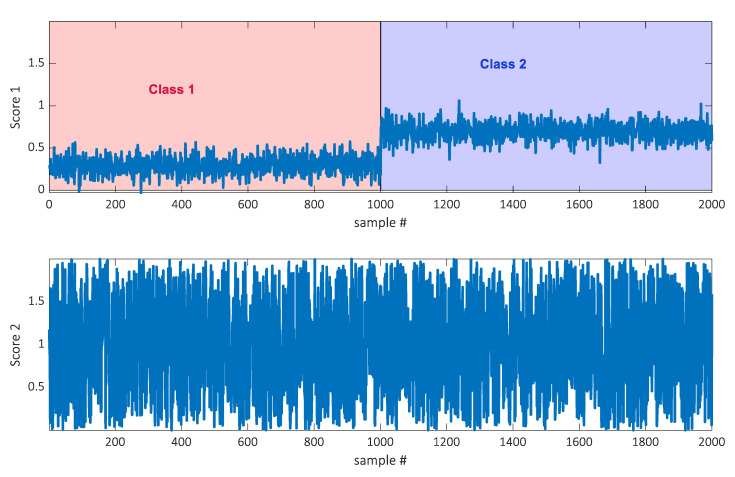
Two scores of two thousand samples from the simulated dataset. Score one indicates the class belongingness.

The datasets were simulated by generating two thousand samples. The data were simulated to be of rank two, which means that a two-component score plot should be able to reflect all information. This was achieved by generating the datasets as a product of two score and two loading vectors. Each sample has two scores as indicated in Figure 1. Corresponding loadings were generated such that the first score vector, the one indicating the class, only loaded on a few variables. Out of 360 variables, 350 had a loading of zero on score 1 and hence only ten variables had a non-zero loading on that component. The ten elements were chosen from a random uniform distribution, with elements between zero and one. The loading vector for score two (with no class information) was defined by drawing the loading elements for all 360 variables from a uniform distribution with elements between zero and one. Hence, most information in the data is not indicative of class belongingness. See Supplementary Materials for a script generating the data. Two thousand samples were generated and every fifty was used for calibration. Hence, the calibration set was size 40 × 360 and the test set was 1960 × 360.

In order to assess the variability of the results, the procedure of generating data, the splitting in calibration and the test and fitting models were repeated 500 times.

### 3.2. Cancer Data

A cancer dataset was taken from an earlier study [10]. A total of 838 blood samples were analyzed. The samples all came from women that were healthy at the time of sampling, but half of the women would be diagnosed with cancer within five years from sampling the blood. The samples were measured by ^1^H NMR and these were integrated into 200 quantified peaks. Hence, the dataset was of size 838 × 200.

In the model building, 240 samples were chosen for calibration and the remaining were used as a test set. In the calibration set, the two classes were represented equally as much, hence there were 120 samples from each class. The test set was also balanced in terms of class. The reason for the quite large test set was simply to make the calibration set small enough to have overfitting. In the original application, the calibration set was much larger and presented no overfitting. The samples were chosen randomly for the calibration set within each class and the remaining samples were put in the test set. As for the simulated data, the procedure of splitting the data and fitting models were repeated 500 times to assess the variability of the results.

## 4. Results

**Figure 2:**
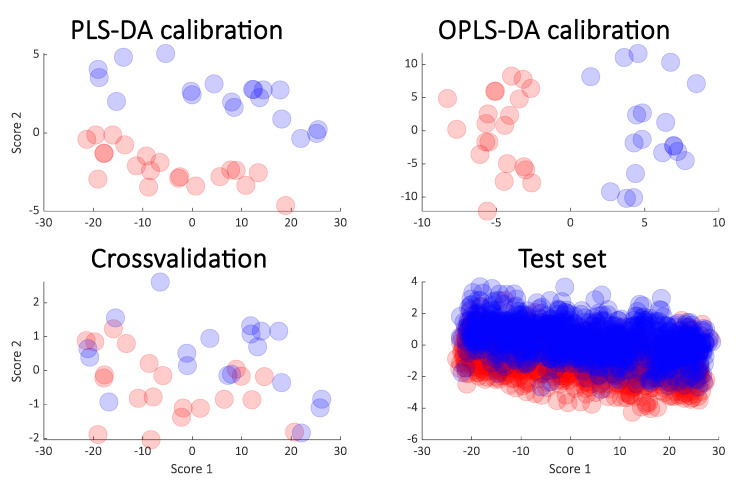
Calibrated score plot from PLS-DA (**upper left**) and the OPLS-DA (orthogonal PLS-DA) (**upper right**) from the simulated data. The cross-validated scores from PLS-DA (**lower left**) and the score plot obtained from the test samples (**lower right**) are also shown.

For the simulated data, the rank was two and a two-component PLS-DA model will therefore be optimal and was chosen here for building the classification. In Figure 2, the resulting score plots are shown. The PLS-DA and OPLS-DA have the same predictive power and for that reason, cross-validation results are only shown for one of them: PLS-DA. The test set results are from the PLS-DA model, too.

**Figure 3:**
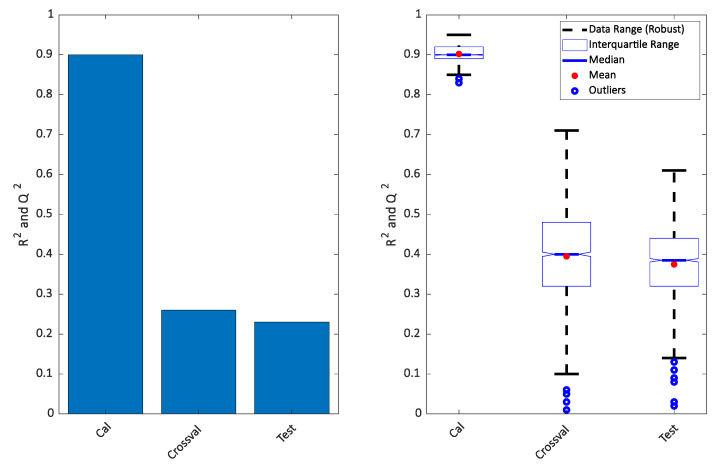
Variance described for the model plotted in Figure 2 (**left**) and repeated 500 times (**right**). The calibrated results of PLS-DA and OPLS-DA will be the same, so only one is shown.

It is clear that the PLS-DA score plot from the calibration model (upper left in the Figure 2) seems to indicate a perfect separation. This is looking even better in the OPLS-DA calibrated score plot (upper right in Figure 2) and it is also reflected in the high *R^2^* seen in Figure 3. However, the corresponding *Q^2^* for both the cross-validation and the test set are telling a different story. The corresponding score plots are consistent with the low *Q^2^* values and show a large overlap of the two groups. The similarity of the two *Q^2^* values and the similarity of the score plots (Figure 2 lower left and right) shows that the cross-validated score plots provide a much better and scientifically more meaningful visualization of the model. In Figure 3 right, the result of re-defining calibration and test is shown. The calibration and test sets were defined 500 times and each time, calibration, cross-validation and test set performance (*R^2^* and *Q^2^*) was assessed.

**Figure 4:**
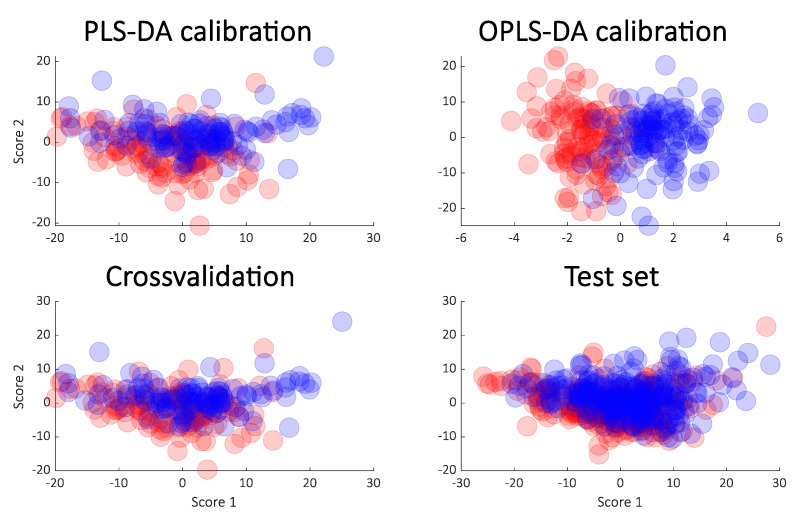
Score plot from PLS-DA (**upper left**) and OPLS-DA (**upper right**) from the cancer data. The cross-validated scores (**lower left**) and the score plot obtained from the test samples (**lower right**) are also shown.

**Figure 5:**
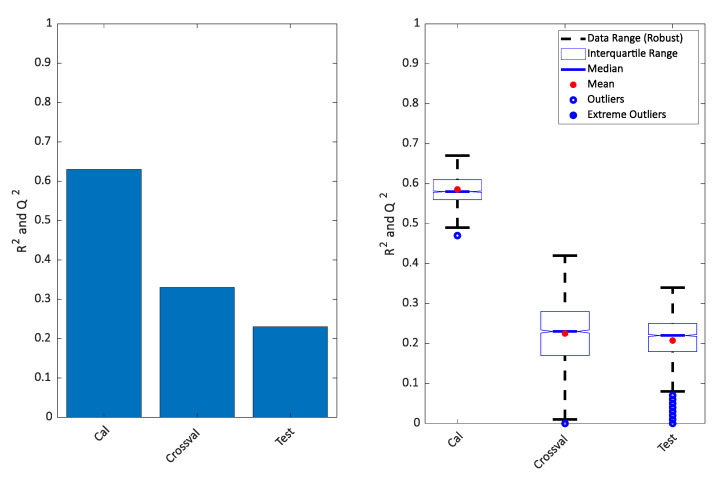
Variance described for the model plotted in Figure 4 (**left**) and repeated 500 times (**right**).

A similar approach was taken on the cancer data. The rank of the PLS models was chosen to be six as in the original publication, and for that reason, a score plot of just components one and two of the PLS-DA model was not necessarily expected to show a clear separation. Indeed, this is the case as seen in Figure 4 (upper left) but the OPLS (upper right) still indicates an almost perfect separation. As for the simulated data, the cross-validation and test sets show a completely different story both in terms of the variances explained (Figure 5) and in the score plots (Figure 4). It is again clear that the calibrated score plots by no means are indicative of the predictive power.

## 5. Conclusions

We showed that when *R^2^* >> *Q^2^*, the score plot of a PLS calibration model can be grossly misleading. This holds for PLS and even more so for OPLS. The problem can be solved completely by using cross-validated scores and we suggest that papers are not accepted when including calibrated scores plots in general, unless the *R^2^* and *Q^2^* are of comparable size.

## Supplementary Materials

The following are available online at https://www.mdpi.com/2218-1989/10/7/278/s1.
